# Smart Responsive Nanoformulation for Targeted Delivery of Active Compounds From Traditional Chinese Medicine

**DOI:** 10.3389/fchem.2020.559159

**Published:** 2020-12-10

**Authors:** Xuejun Jiang, Mei Lin, Jianwen Huang, Mulan Mo, Houhe Liu, Yuan Jiang, Xiaowen Cai, Wingnang Leung, Chuanshan Xu

**Affiliations:** ^1^Key Laboratory of Molecular Target and Clinical Pharmacology, State Key Laboratory of Respiratory Disease, School of Pharmaceutical Sciences & Fifth Affiliated Hospital, Guangzhou Medical University, Guangzhou, China; ^2^Asia-Pacific Institute of Aging Studies, Lingnan University, Hong Kong, China

**Keywords:** smart responsive nanoformulation, traditional Chinese medicine, targeted drug delivery, tumor, infectious disease

## Abstract

Traditional Chinese medicine (TCM) has been used to treat disorders in China for ~1,000 years. Growing evidence has shown that the active ingredients from TCM have antibacterial, antiproliferative, antioxidant, and apoptosis-inducing features. However, poor solubility and low bioavailability limit clinical application of active compounds from TCM. “Nanoformulations” (NFs) are novel and advanced drug-delivery systems. They show promise for improving the solubility and bioavailability of drugs. In particular, “smart responsive NFs” can respond to the special external and internal stimuli in targeted sites to release loaded drugs, which enables them to control the release of drug within target tissues. Recent studies have demonstrated that smart responsive NFs can achieve targeted release of active compounds from TCM at disease sites to increase their concentrations in diseased tissues and reduce the number of adverse effects. Here, we review “internal stimulus–responsive NFs” (based on pH and redox status) and “external stimulus–responsive NFs” (based on light and magnetic fields) and focus on their application for active compounds from TCM against tumors and infectious diseases, to further boost the development of TCM in modern medicine.

## Introduction

Traditional Chinese medicine (TCM) as an important approach to treat disorders has been used widely in China for ~1,000 years. Accumulating evidence has shown that TCM exhibits excellent effects on tumors (Luo et al., 2019), bacteria (Kim et al., 2008; Moloney, 2016; Wang Z. et al., 2018b), and viruses (Li et al., 2018a; Yao et al., 2018; Yang et al., 2020) because of their active compounds. Some active compounds, such as artemisinin, curcumin, and epigallocatechin gallate, emodin, and celastrol, show prominent efficacy against tumors (Wang et al., 2014; Shanmugam et al., 2017). Flavonoids, polyphenols, alkaloids, and terpenoids are considered to be efficacious against antibiotic-resistant bacteria (Zhao et al., 2019b). Also, TCM shows multitarget features against viruses in direct or indirect ways (Ai et al., 2018; Li et al., 2018a).

However, the poor solubility and low bioavailability of the active compounds from TCM limit their clinical application. To address these shortcomings, novel drug-delivery nanoformulations (NFs) are being employed to increase solubility, improve the efficiency of targeted delivery, and reduce the number of adverse effects. Recently, liposomes, nanoparticles, vesicles, mesoporous silica nanoparticles, and micelles as potential drug-delivery NFs have shown great promise in TCM (Ma et al., 2019).

**Figure 1:**
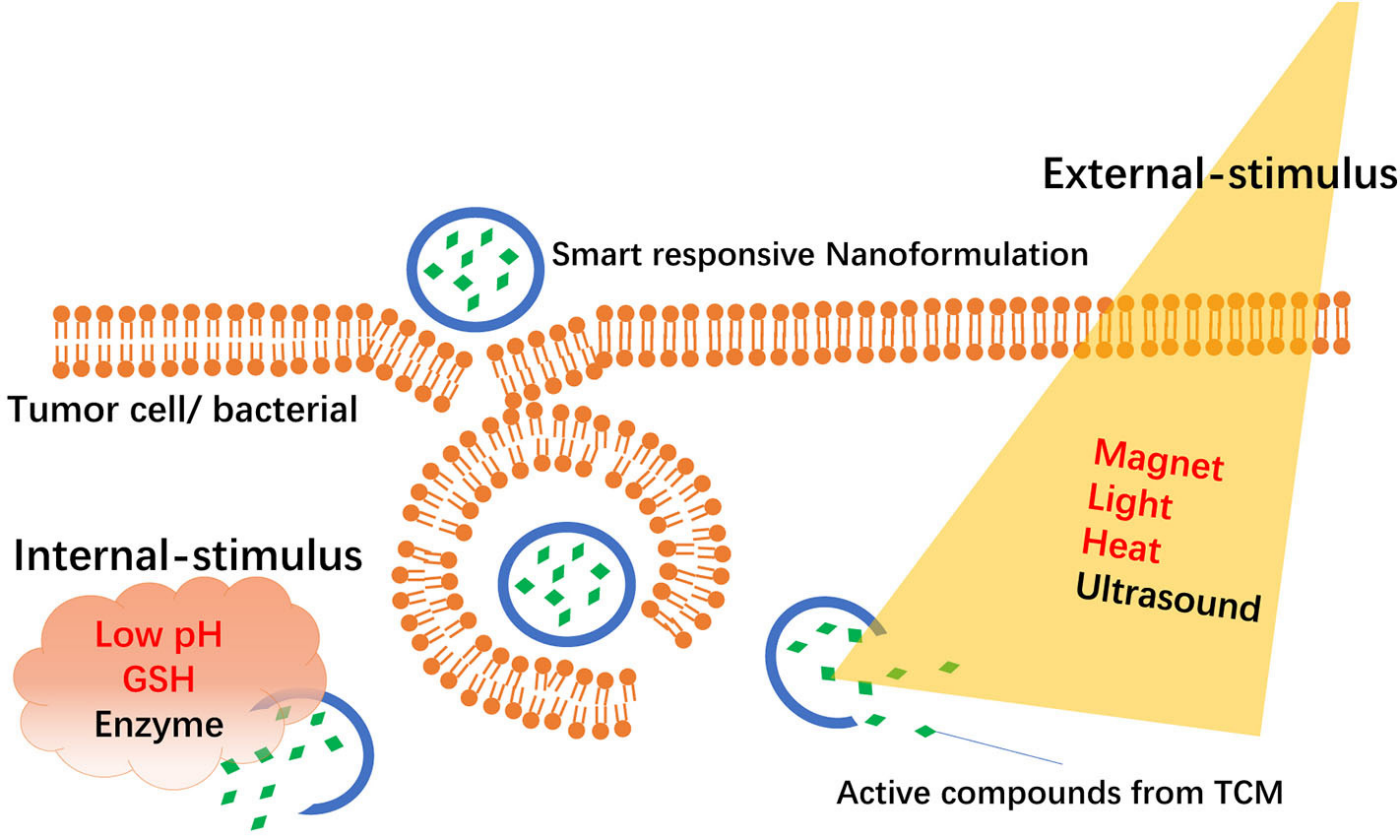
Schematic representation of smart responsive nanoformulations-mediated delivery of active compounds from TCM.

“Smart responsive NFs” are new types of targeted NFs that deliver drugs specifically to target tissues or organs *via* an “intelligent response” in target sites. Usually, smart responsive NFs are classified into “internal stimuli–responsive NFs” and “external stimuli–responsive NFs” according to different stimuli conditions as shown in Figure 1. Internal stimuli–responsive NFs are mainly from the microenvironment of the targeted site and include pH, enzymes, redox status, and receptors. External stimuli–responsive NFs are mainly from magnets, heat, light, or ultrasound.

Here, we reviewed internal stimulus–responsive NFs and external stimulus–responsive NFs. We focused on the application of active compounds from TCM against tumors and infectious diseases, to further boost the development of TCM concepts in modern medicine.

## Internal Stimulus–Responsive NFs

### pH-Responsive NFs for Delivery of Active Compounds From TCM

The metabolism of tumor cells involves aerobic glycolysis to obtain energy for overgrowth and proliferation (Ganapathy-Kanniappan and Geschwind, 2013). Aerobic glycolysis is based on the conversion of glucose to pyruvate, which results in fermentation and lactate production (Shi et al., 2018), and the resulting acidosis is a ubiquitous characteristic of cancer (Tang et al., 2018). It has been demonstrated that the tumor tissues' acidic environment is about pH 6.5 (Som et al., 2016), whereas the pH of normal tissue is 7.4 (Liu et al., 2014). The microenvironment of tissues infected by bacteria is also acidic (Tao et al., 2019a). As shown in Table 1, based on the characteristics of an acidic microenvironment, pH-responsive NFs have been developed for targeted delivery of active compounds from TCM.

**Table 1:** pH-responsive nanoformulations in active compounds from TCM.

**Active compounds**	**Response agent**	**Application**	**Cell line/animal**	**References**
Artemisinin	Fe_3_O_4_ nanocrystal	Cervical cancer	HeLa	Chen et al., 2014
	Fe_3_O_4_@MnSiO_3_	Lung cancer	A549 Nude mice	Chen et al., 2015
	Fe_3_O_4_ nanoparticles	Breast cancer	ZR75-30	Bhaw-Luximon and Jhurry, 2017
Arsenic trioxide	Polyamidoamine	Glioma	C6	Lu et al., 2018
Dihydroartemisinin	Fe (III)	Breast cancer	EMT6 EMT6 tumor-bearing nude mice	Liu et al., 2015
Baicalein	Hydrazone bond	Lung cancer	A549/DTX cell A549/DTX tumor-bearing nude mice	Li et al., 2017
Berberine	Chitosan	*H. pylori* infection		Lin et al., 2015
	Amine bond	Cervical cancer	HeLa	Pandey et al., 2013
Cinnamaldehyde	Acetal	Anticancer	DU145 SW620 RAW264.7 HEK 293 BALB/c nude mice	Yoo et al., 2018
	Acetal	*E. coli* infection *P. aeruginosa* infection	*E. coli* Drug-resistant *P. aeruginosa* DRPA-001 ICR mice	Park et al., 2016
	Acetal	Colon cancer	HCT116 BALB/cA nu/nu mice	Zhao et al., 2019a
Curcumin	PAE	Breast cancer	MCF-7 BALB/c nude mice	Yu et al., 2015
	Amine bond	Liver cancer	HepG2	Luan et al., 2017
	ZnO nanoparticle	Gastric cancer	AGS	Dhivya et al., 2018
	PAE	Hepatocellular carcinoma	SMMC 7721 HUVEC BALB/c nude mice	Zhang et al., 2017
	PAE	Anticancer	MCF-7 HepG2	Cai et al., 2016
	*cis*-Aconitic anhydride linker	Anticancer	A2780 SMMC 7721	Fang et al., 2016
	Poly(isoprene-b-acrylic acid) deblock copolymer	Acute myelocyte leukemia	HL-60 HL-60/Dox HL-60/CDDP	Jelezova et al., 2015
	Carboxylic groups of AGA	Colorectal cancer	HCT-116	Madhusudana Rao et al., 2015
	Menthone 1,2-glycerol ketal	Anticancer	SCC-7 tumor-bearing BALB/c mice	Chen et al., 2016a
	Hydrazone bond	Breast cancer	MCF-7 BALB/c nude mice	Cui et al., 2017
	Hydrazone bond	Breast cancer	MCF-7 Nude mice	Liu et al., 2019
	Calcium phosphate	Anticancer	MGC-803 A549	Wu et al., 2020
Eucalyptus oil	PLGA	Skin cancer	HaCaT	Sahu et al., 2017
Glycyrrhetinic acid	Chitosan	Liver cancer	QGY-7703 BALB/c nude mice Wistar rats	Tian et al., 2010
Glycyrrhetic acid	Hydrazone bond	Liver cancer	HepG2 HUVEC	Yan et al., 2018
Physcion	ZIF-8	Antibacteria	*Pseudomonas putida* *Escherichia coli* Engineered *Escherichia coli* *Staphylococcus aureus*	Soomro et al., 2019
Myricetin Quercetin Fisetin Luteolin	Coordination bond	Antibacteria	—	Bertleff-Zieschang et al., 2017
Quercetin	PAA	Anticancer	—	Sedghi et al., 2013
	PLGA	Skin cancer	HaCaT A431	Gupta et al., 2016
	*N*-acetyl-l-histidine	Anticancer	NIH/3T3 Red blood cell	Wu et al., 2016
	Boronic ester bond	Anticancer	MCF-7 A549 HepG2 IRC mice	Xing et al., 2017
	Polydopamine	Multidrug-resistant colon cancer	HCT-8 HCT-8/TAX	Shao et al., 2019
	Amide bond and ionic bond	Multidrug-resistant	MCF-7 MCF-7/DOX	Liu et al., 2017
	Chitosan	Anticancer	MCF-7	de Oliveira Pedro et al., 2018
Triptolide	Ionizable imidazole side chains	Hepatocellular carcinoma	Bel-7404 HCCLM3 Old athymic mice	Ling et al., 2014
	Tertiary amine group	Pancreatic cancer	PANC-1 MIA PaCa-2 BALB/C nude mice	Kong et al., 2019
	Silk	Pancreatic cancer	MIA PaCa-2 PANC-1	Ding et al., 2017
Ursolic acid	Chitosan	Anticancer	HeLa CD-1 female mice	Wang M. et al., 2017

#### Delivery of Artemisinin and Dihydroartemisinin

Artemisinin and dihydroartemisinin are active agents from the Chinese herb *Artemisia annua*. They are used as antimalarial drugs in China (Tu, 2016). They are also potential anticancer agents when interacting with iron ions to produce reactive oxygen species (ROS), which can kill malignant cancer cells (Efferth, 2017). Chen et al. developed Fe_3_O_4_ nanoparticles as a pH-sensitive NF, for treating cervical cancer (Chen et al., 2014). In this NF, artemisinin was stored in the outer mesoporous shells. Fe^2+^ was liberated from the core of the Fe_3_O_4_ nanosphere in acidic organelles and cleaved the endoperoxide bridges of artemisinin to generate free radicals to kill HeLa cells. Artemisinin-hollow mesoporous silica-Fe_3_O_4_ nanoparticles have also been used to treat breast cancer *via* pH-responsive Fe_3_O_4_ nanoparticles (Bhaw-Luximon and Jhurry, 2017). Sustained release of artemisinin occurred in the lysosomal compartment (pH 3.8–5.0), and Fe_3_O_4_ was metabolized to free Fe^2+^ to generate many free radicals through reaction with the released artemisinin. In addition, an Fe_3_O_4_@MnSiO_3_-folate nanosphere was developed for the treatment of lung cancer (Chen et al., 2015). Folate was introduced to increase the enhanced permeability and retention (EPR) effect. Meanwhile, Mn^2+^ was released from the silicate shells and interacted with artemisinin in an acidic tumor environment and organelles of A549 cells.

Liu et al. (2015) prepared dihydroartemisinin–graphene oxide–transferrin nanoparticles. The Fe (III) conjugated on transferrin was released in lysosomes and was reduced to Fe (II) by ferric reductase. Fe (II) reacted with dihydroartemisinin on graphene oxide to yield ROS and oxygen/carbon-centered radicals, which then induced the death of EMT6 cells.

#### Delivery of Baicalein

Baicalein is a flavonoid from *Scutellaria baicalensis* Georgi. which has antioxidant, antivirus, antibacterial, anti-inflammatory, and antiallergic properties (Bie et al., 2017). Baicalein can inhibit the cell cycle; scavenge oxygen radicals; attenuate the activity of mitogen-activated protein kinase, protein kinase B, or mammalian target of rapamycin signaling pathways; and induce apoptosis by activating caspase-9/-3 (Liu et al., 2016). Recently, baicalein was delivered by a pH-responsive cleaved hydrazone bond in transferrin decorated docetaxel baicalein co-loaded solid lipid nanoparticles (Li et al., 2017). A modification based on polyethylene glycol (PEG) provided this system a long circulation in the body. Also, transferrin could guide delivery of nanoparticles into tumor cells by transferrin receptors. At an acidic condition, the hydrazone bond ruptured, resulting in shedding of the PEG layer from nanoparticles and the targeted release of baicalein.

#### Delivery of Cinnamaldehyde

Cinnamon is used not only as a spice, but also in TCM. Cinnamaldehyde is an active compound in cinnamon that can induce the apoptosis of tumor cells (Sadeghi et al., 2019). Yoo et al. (2018) used acetal linkages to couple cinnamaldehyde and maltodextrin to fabricate a cinnamaldehyde-maltodextrin nanoparticle. The acetal linkage dissociated in the acidic environment, and cinnamaldehyde and maltodextrin could return to their native states. Zhao et al. (2019a) also used acetal linkages to couple cinnamaldehyde and dextran in 10-hydroxy camptothecin-cinnamaldehyde nanoparticles. The acetal linkage of cinnamaldehyde was hydrolyzed from dextran at an acidic pH, which broke the nanoparticles into water-soluble fragments, and resulted in rapid dissociation. Ferrocene-loaded poly[(3-phenylprop-2-ene-1,1-diyl)bis(oxy)bis(ethane-2,1-diyl)diacrylate]-co-4,4′(trimethylene dipiperidine)-copoly(ethylene glycol) micelles have been designed to release ferrocene and cinnamaldehyde rapidly at the site of bacterial infections (which are characterized by a low pH) (Park et al., 2016). Cinnamaldehyde induced the generation of hydrogen peroxide (H_2_O_2_) and iron in ferrocene and then converted H_2_O_2_ into highly toxic hydroxyl radicals.

#### Delivery of Curcumin

Curcumin is a polyphenol extracted from the herb turmeric. It has gained attention worldwide because of its antioxidant, anti-inflammatory, antimicrobial, and antiviral activities (Giordano, 2019). NF development can circumvent the poor bioavailability of curcumin to improve treatment outcome (Adiwidjaja and McLachlan, 2017).

Poly(β-amino ester) (PAE) is a typical pH-responsive biodegradable polymer. PAE has outstanding characteristics, such as positive charges, readily degradable linkages, high biodegradability, and high biocompatibility (Cordeiro et al., 2019). Yu et al. developed poly(ethylene glycol)-poly(lactide)-PAE micelles to deliver curcumin to the breast cancer cell line MCF-7. PAE is insoluble at pH 7.4 and maintains a large micelle structure with a hydrophobic PLA/PAE core (Yu and Zhang, 2014). However, PAE is soluble at pH 6.8 (thanks to protonation of tertiary amino groups) and shrinks the micelle structure to a hydrophobic PLA core. d-α-Tocopheryl PEG 1000-block-PAE (TPGSPAE) nanoparticles used in treatment of hepatocellular carcinoma have a similar mechanism of action (Zhang et al., 2017). The tertiary diamine moieties of the PAE core are protonated at low pH so that the hydrophobic curcumin is released from the hydrophilic core-shell structure. In curcumin-pluronic P123-PAE (Cai et al., 2016), P123 is constituted by poly(ethylene oxide)-poly(phenylene oxide)-poly(ethylene oxide) (PEO-PPO-PEO). The PPO/PAE core offers a local hydrophobic microenvironment for curcumin loading, and the hydrophilic PEO shell maintains a large micelle structure at pH 7.4. PAE is protonated and dissolves at weakly acidic pH, making the nanocarrier shrink to be a small micelle structure and release curcumin.

pH-sensitive linkages have also been used for curcumin delivery. At pH 4.5, chains of β-acrylic acid undergo transition to the protonated state. This action lowers their solubility abruptly and isolates them from the aqueous phase and results in rearrangements of the bilayer membrane and curcumin leakage. Fang et al. (2016) coupled a poloxamer (F68) and curcumin by a *cis*-aconitic anhydride linker. In acidic environments, the pH-sensitive *cis*-aconitic anhydride linker of F68-*cis*- curcumin conjugates were cleaved to release curcumin. Chen et al. (2016a) reported a menthone 1,2-glycerol ketal (MGK), which was used in Cur-hyaluronan-histidine-MGK. The ketal moieties of MGK could be degraded in a tumor microenvironment. Also, the imidazole group of histidine, which linked oligomeric hyaluronic acid (oHA) and MGK, could destroy lysosomal membranes to prevent the drug being degraded in cells. Luan et al. found that an acidic condition (pH < 6.5) could cause breakage of internal linkages in aliphatic amines grafted konjac glucomannan (KGM-g-AH) between a primary amine of octylamine and an aldehyde group and release curcumin. The responsive agent in KGM-g-AH8 micelles was KGM (Luan et al., 2017). Similarly, pH-sensitive hydrazone bonds have been used to deliver curcumin. Polymer oHA-hydrazone bond-folic acid biotin nanomicelles consisting of folic acid, biotin, and cluster of differentiation (CD)44 receptors can mediate the targeting of tumor tissue and cancer stem cells, from which the icariin and curcumin are released into the tumor microenvironment, depending on the pH-sensitive hydrazone bond (Liu et al., 2019).

Eucalyptus from *Eucalyptus robusta* Smith has been developed as a transdermal delivery vehicle for curcumin (Liu and Chang, 2011). Sahu et al. reported a 5-fluorouracil double-walled nanogel (FDWNL) to load eucalyptus with pH-sensitive chitosan. The hydrophilic pendant groups of chitosan (-OH and -NHCOCH_3_) and strong ionic attraction between the ionized nanogel and hydrated counter ions caused rapid swelling of the FDWNL to release curcumin at acidic pH (Sahu et al., 2017).

Among metal nanoparticles, ZnO nanoparticles are pH-sensitive nanoplatforms used for curcumin delivery. The nontoxic ZnO nanoparticles are stable at pH ~7, but dissolve and open up the polymer linkages of polymethyl methacrylate-acrylic acid ZnO nanoparticles to release curcumin together with toxic Zn^2+^ at a low pH (~5.4) (Dhivya et al., 2018). Curcumin was also connected to one side of the disulfide-condensed menthone 1,2 glycerol ketal as a redox response prodrug material. The other side of menthone 1,2 glycerol ketal was connected to hyaluronic acid with an ester bond that made pH/redox response. The pH-responsive calcium phosphate shell made this NF broken at pH 5.5 to release curcumin to kill cancer (Chen et al., 2017). Wu et al. also used calcium phosphate as a pH-responsive shell to wrap the curcumin-adsorbed sodium caseinate micelles to improve the stability of curcumin. In the study, Wu et al. found that the calcium phosphate shell covering the sodium caseinate micelles was broken in the acidic stroma outside the tumor cells (Wu et al., 2020).

#### Delivery of Glycyrrhetinic Acid

Glycyrrhetinic acid (GA) is extracted from licorice and is modified for targeting of liver cells (Zhu et al., 2019, 2020; Li et al., 2020). In doxorubicin-loaded chitosan/poly(ethylene glycol)-GA nanoparticles, GA increases the affinity for liver cancer cells (Tian et al., 2010). Moreover, protonation of the amino groups of chitosan at an acidic condition and the acid-soluble doxorubicin hydrochloride cause the swelling of nanoparticles and release doxorubicin. In GA-modified chitosan-polyethyleneimine-4-hydrazinobenzoic acid–doxorubicin nanoparticles, GA improves the liver-targeting ability, and the hydrazone bond between doxorubicin and GA-CS-PEI-HBA is broken to release the “payload” in the intracellular environment (pH 4.5–6.5) of tumor cells (Yan et al., 2018).

#### Delivery of Triptolide and Celastrol

Triptolide and celastrol are contained in *Tripterygium wilfordii*. Triptolide has excellent inhibitory effects in the treatment of pancreatic cancer (Kim et al., 2018). Kong et al. (2019) enhanced the efficacy of triptolide by developing triptolide prodrug-loaded ultra–pH-sensitive micelles (T-UPSMs). After internalization by the endocytic organelles of cancer cells, the pH reached the apparent acid dissociation constant of T-UPSMs due to lysosomal acidification. The tertiary amine groups of T-UPSMs absorbed protons and induced micelle dissociation to elicit immediate drug release as a pH buffer in endosomes/lysosomes. Ding et al. (2017) found that triptolide or celastrol was weakly adsorbed or bonded to silk fibroin nanoparticles. At a low pH, silk loses its overall acidic surface properties and negative net charge, and the balance of the negative charge is shielded at an acidic pH, which destroys aggregates. Ling et al. (2014) developed nanoformulated triptolide nanoparticles for treatment of hepatocellular carcinoma. Internally packed imidazole was employed for pH-sensitive ionization and dispersion. During endosomal maturation, the decrease in pH could lead to the collapse of nanoparticles and triptolide release.

#### Delivery of Ursolic Acid

Ursolic acid (UA) is present in *Prunella vulgaris L*. but is also abundant in most fruits and vegetables (Yin et al., 2016). UA use has been reported in treatment of cancer (Jaman, 2018; Manayi et al., 2018; Mlala et al., 2019) and some infections (Tohmé et al., 2019). UA has been encapsulated in nanoparticles or liposomes and found to inhibit progression of cervical cancer (Wang M. et al., 2017; Wang S. et al., 2017). Jiang et al. (2017) prepared UA-loaded mesoporous silica nanoparticles and folic acid conjugated chitosan (UA@M-CS-FA) nanoparticles in which the acid-labile amide bond between CS-FA and mesoporous silica nanoparticles was broken in acidic conditions. This action made the chitosan chains swell and opened the mesopores of UA@M-CS-FA nanoparticles, resulting in UA release.

#### Delivery of Berberine

Berberine is isolated from *Coptis chinensis* and has antibacterial effects. Berberine was shown to markedly increase the survival rate of mice challenged with the bacterial endotoxin lipopolysaccharide (2 EU/mL) (Chu et al., 2014). Chitosan and heparin are ionized and form polyelectrolyte complexes *via* electrostatic interactions at pH 1.2–6.0 to protect berberine from destruction by gastric acids. At pH 7.0, chitosan is deprotonated, and berberine-loaded fucose-conjugated nanoparticles are broken apart to release berberine (Lin et al., 2015). Moreover, berberine as a hydrophobic part was linked to chitosan oligosaccharides with a dithiodipropionic acid linker, and 3,4-dihydroxyphenylpropionic acid as a hydrophilic part was also linked to another end of the chitosan oligosaccharides to form a polymer monomer. The positively charged 3,4-dihydroxyphenylpropionic acid attracted the negatively charged hyaluronic acid to form an oHA-3-carboxyphenylboronic acid shell and encapsulate curcumin. In the acidic microenvironment of tumor, the exposed hyaluronic acid specifically binds to the CD44 receptor, and the micelles are endocytosed by the cells and undergo charge reversal. The high concentration of glutathione (GSH) in tumor cells hydrolyzes the disulfide bonds of dithiodipropionic acid; the micelles collapse and release berberine and curcumin (Fang et al., 2019b).

#### Delivery of Physcion

Usually, zeolitic imidazolate framework-8 (ZIF-8) is fabricated using zinc ions and 2-methylimidazolate. ZIF-8 has high porosity, good structural regularity and tunability, adjustable surface functionality, and intrinsic pH-induced biodegradability (Gao et al., 2019b). Soomro et al. (2019) developed physcion@ZIF-8 to deliver the physcion present in rhubarb. In acidic conditions, the coordination linkage between zinc and imidazolate was broken, whereas the imidazolate was protonated, resulting in physcion release from ZIF-8.

#### Delivery of Quercetin

Quercetin from *Hypericum ascyron L*. has been reported to be beneficial for cardiovascular disease (Patel et al., 2018). In recent years, the nanotechnology transformation of quercetin has revealed that quercetin has considerable effects against tumors (Vinayak, 2019). Bertleff-Zieschang et al. reported quercetin/Fe (III) nanoparticles as potential antibacterial NFs. In that work, formation of a quercetin/Fe (III) network was pH-dependent based on coordination between a flavonoid ligand and Fe (III). At an acidic pH, quercetin/Fe (III) capsules were disassembled readily (Bertleff-Zieschang et al., 2017). Furthermore, amphiphilic pH-responsive 8-arm- and 12-arm-dendritic polyacrylic acid block copolymers were synthesized for quercetin loading. *In vitro* release experiments showed that both of these new materials achieved complete release faster at pH 5.0 than at pH 7.4. But the 12-arm dendritic polyacrylic acid polymer blocks copolymer release quercetin faster than the 8-arm-one (Sedghi et al., 2013). In a self-assembled micelle of *N*-acetyl-histidine-phosphocholine-chitosan conjugate, histidine was a pH-responsive molecule because of its imidazole group. At pH ≤ 6.0, the micelles swelled and ruptured to release the encaspsulated quercetin due to the protonation of the imidazole group (Wu et al., 2016).

### Redox-Responsive NFs

GSH depletion promotes cancer cell death through processes such as apoptosis, necroptosis, autophagy, and ferroptosis (Lv et al., 2019). GSH deficiency, or a decrease in the ratio of GSH and glutathione disulfide (GSSG), leads to an increased susceptibility to oxidative stress implicated in the progression of cancer cells (Traverso et al., 2013). The GSH:GSSG ratio, as the major pool of thiol groups, is a key factor in the antioxidative capacity of cells. GSH is an ideal and omnipresent internal stimulus for rapid degradation of disulfide linkages (Zhang et al., 2018b). As shown in Table 2, there are many applications of disulfide bonds in redox-responsive delivery of TCM agents.

**Table 2:** Redox-responsive nanoformulations in active compounds from TCM.

**Active compounds**	**Response agent**	**Application**	**Cell line/animal**	**References**
Eucalyptus oil	Disulfide bond in 3,30-dithiodipropionic acid	Lung cancer	A549 RAW264.7 A549 bearing mice	Wang et al., 2019a
Baicalin Curcumin Quercetin Ferulic acid	Dithiodipropionic acid–Cur	Anticancer	HepG2 A549 tumor-bearing mice	Wang K. et al., 2018
Curcumin	Cystamine	Glioma	G422	Tian et al., 2018
	Disulfide bond	Anticancer	MDA-MB-231	Dong et al., 2018
	Disulfide bond	Anticancer	HepG2 BALB/c mice	Zhang et al., 2019
Glycyrrhetic acid	PLGA	Lung cancer	Beas2B A549 NCI-H226	Zhang et al., 2020
Homoharringtonine	PLGA	Lung cancer	Beas2B A549 NCI-H226 BALB/c-nude mice	Zhang et al., 2020
Oridonin	Oxalate ester bond	Colon carcinoma	CT26	Ou et al., 2019
Quercetin	Disulfide bond	Multidrug-resistant	4T1 L-02 BALB/C mice	Chen et al., 2018

Wang B et al. (2019) designed quercetin–dithiodipropionic acid–oHA–mannose–ferulic acid “nano-dandelions” for synchronous delivery of curcumin and baicalein. This was achieved by using the nano-dandelions as reduction-sensitive amphiphilic carriers. The coated oHA targeted CD44 receptors and could facilitate uptake of nano-dandelions in tumor locations. Mannose-targeting CD206 receptors could be engulfed readily by tumor-associated macrophages. The S-S linkage in 3,30-dithiodipropionic acid connecting the hydrophobic and hydrophilic parts could be broken by the high concentration of GSH within tumor cells, which facilitated release of curcumin and baicalein.

Wang K. et al. (2018) developed distearoyl phosphatidyl ethanolamine derivatized PEG-modified nano-echinus materials. Dithiodipropionic acid–curcumin (S-S-Cur) was chemically conjugated onto the side chain of the conjugated GA-oHA (GA-HA) to generate an amphiphilic polymeric prodrug of curcumin (GA-HA-S-S-Cur). HA targeted CD44 receptors on the surface of tumor cells, and the breaking of disulfide bonds by a high concentration of GSH in a tumor environment led to disassembly of nano-echinus materials, and then released curcumin. Tian et al. (2018) developed HA-S-S-CUR micelles for glioma treatment, in which the disulfide bond of cystamine linked HA (hydrophilic group) and curcumin (hydrophobic group). Reductive cleavage of the disulfide bond by GSH in glioma cells caused instability of the hydrophobic core, resulting in micelle degradation and curcumin release.

In alendronate-oHA-S-S-curcumin micelles (Dong et al., 2018), a disulfide bond combines hydrophobic curcumin with hydrophilic oHA and alendronate to form micelles in water. These micelles fracture under a reducing environment to release curcumin. Cleavage of the disulfide bonds of PEGylated prodrug nanomicelles (Zhang et al., 2019) can transfer these nanomicelles into hydrophilic curcumin-mercapto. This action accelerates hydrolysis of the adjoining ester bond and releases curcumin from PEGylated prodrug nanomicelles.

Recently, synthetically designed polymers containing disulfide bonds have been used to deliver TCM-active compounds. Homoharringtonine (HHT)–loaded poly(lactic-co-glycolic acid)-SS-PEG (Zhang et al., 2020a) is an epidermal growth factor receptor (EGFR) aptamer-modified PLGA targeted to the EGFR (which shows high expression in lung cancer cells). The drug is delivered into the cytoplasm *via* receptor-mediated endocytosis. The disulfide bonds of PLGA are broken by GSH in lung cancer cells.

Additionally, ester bonds have also been used in the development of redox-responsive NFs. In the podophyllotoxin (POD) delivery, PODPEG nanoparticles (Ou et al., 2019) are formed by PEG and POD with oxalate ester bond bridges. The latter are cleaved in the presence of H_2_O_2_ to release the drug.

## External Stimulus–Responsive NFs

External stimuli–responsive NFs for delivery of active compounds from TCM are mainly including magnetic, light, and thermal response as shown in Table 3.

**Table 3:** External stimulus–responsive nanoformulations in active compounds from TCM.

**Response method**	**Active compounds**	**Response agent**	**Application**	**Cell line/animal**	**References**
Magnetic	Artemisinin	Magnetic iron oxide	Breast cancer	BALB/c mice	Gharib et al., 2015
	Dihydroartemisinin	Fe_3_O_4_ nanoparticle	Head and neck squamous cell carcinoma	A549 HeLa HeLa tumor-bearing nude mice	Li et al., 2019a
		Fe_3_O_4_ nanoparticle	Myeloid leukemia	K562 HL-60 SHI-1 NB4 BALB/c male mice	Chen et al., 2016b
	Quercetin	γ-Fe_2_O_3_	Glioma	C6	Cruz Dos Santos et al., 2019
	Stevioside	Fe_3_O_4_	Lung cancer	A549	Gupta and Sharma, 2019
Thermal	Curcumin	Poly(*N*-isopropylacrylamide)	Anticancer	L929 KB MCF-7 PC3	Rejinold et al., 2011
	Honokiol	(PEG-PCL-PEG, PECE) hydrogel	Malignant pleural effusion	LL2 Red blood cell C57B/6 mice	Fang et al., 2009
Light	Artesunate	Covalent bond between carboxylic groups and amino groups of nGO-PEG	Liver cancer	HepG2 4T1 tumor-bearing mice	Pang et al., 2017
	Aloe emodin	Aloe emodin	Gastric cancer	SGC-7901	Li et al., 2016
	Hypericin	Hypericin	Glioma	U87-MG	Huntosova et al., 2012
		Hypericin	Colon carcinoma Antibacterial	Caco-2 HT-29 *E. faecalis* *E. coli* *S. aureus*	Montanha et al., 2017
		Hypericin	Ovarian cancer	A2780N A2780N tumor-bearing nude mouse	Han et al., 2020
	Quercetin	Gold nanocages	Multidrug-resistant breast cancer	MCF-7/ADR	Zhang et al., 2018c
	Tetrandrine	Fe-GA	Anticancer	4T1 U87MG	Wang et al., 2019b

### Magnetic-Responsive NFs for Delivery of Active Compounds From TCM

Magnetic-responsive NFs can be used to monitor tumors *in vivo* by noninvasive magnetic resonance imaging (MRI) (Kang et al., 2017). In this way, they can deliver chemotherapy drugs, small-molecule agents, photosensitizers, and small interfering RNA molecules (Zhu et al., 2017). Iron oxide nanoparticles are essential in magnetic-responsive NFs (Vangijzegem et al., 2019).

Gharib et al. created artemisinin- and transferrin-loaded magnetic nanoliposomes coated with iron oxide. The external magnet achieved high concentrations of artemisinin and transferrin in tumors to enhance the antitumor effect of artemisinin in BALB/c mice (Gharib et al., 2015). HHT- Fe_3_O_4_ magnetic nanoparticles were designed to deliver the HHT to tumor sites. HHT inhibited the synthesis of the short-lived protein Mcl-1 by targeting the A-site cleft of eukaryotic ribosomes (Chen et al., 2016b).

Dihydroartemisinin-MLP nanoliposomes were created using Fe_3_O_4_ nanoparticles. The negative charge of dihydroartemisinin-MLPs nanoliposomes under the action of a magnetic field delivered dihydroartemisinin with nonspecific binding to plasma proteins (Li et al., 2019a). Tetrandrine–Fe_3_O_4_-PLGA nanoparticles were used to direct tetrandrine to specific sites upon manipulation by an external magnetic field to increase the tetrandrine concentration. Tetrandrine is derived from *Stephania tetrandra* and is an active compound of a TCM called “Fangji” (Wang K et al., 2019).

In the design of magnetic-responsive nanoliposomes to deliver quercetin, quercetin was not only loaded as an antitumor flavonoid drug and also has strong antioxidant properties to prevent the nanomagnetic liposomes from being oxidized. However, the incorporation of quercetin might weaken the magnetic properties of the nanoliposomes, and it was necessary to precisely control the ratio of quercetin to ensure that the superparamagnetism of the magnetic liposomes was maintained (Cruz Dos Santos et al., 2019).

### Light-Responsive NFs for Delivery of Active Compounds From TCM

NFs can be activated by light (visible, ultraviolet, infrared) for using in photodynamic imaging, photodynamic therapy (PDT) or photothermal therapy (PTT) (Rkein, 2014; Chen and Zhao, 2018). Light-responsive agents (“photosensitizers”) play an important part in such light-responsive NFs (Reeßing and Szymanski, 2017).

Traditional photosensitizers and TCM agents can be co-encapsulated, or a TCM agent can be encapsulated in light-responsive inorganic/organic nanoparticles. Moreover, some active compounds of TCM can be used as photosensitizers to carry out PDT or PTT after light activation.

An artesunate-modified PEGylated nanographene oxide (nGO-PEG-ARS) (Pang et al., 2017) delivered artesunate to liver cancer with the light-responsive agent. The light responsive agent was a covalent bond between carboxylic groups and amino groups of nGO-PEG. Near-infrared irradiation triggered the artesunate loaded on nGO-PEG to produce nitric oxide and peroxynitrite groups to achieve a synergistic chemophotothermal anticancer effect.

In Fe–gallic acid–PEG coordination polymer-based nanoparticles, ultrasmall (5 nm) Fe–gallic acid nanoparticles were formed by mixing FeCl_3_ solution and gallic acid solution (Jin et al., 2017). Surface modification of PEG increased its passive accumulation in tumor cells. Also, the temperature was increased by 20°C after laser irradiation for 5 min (808-nm laser at 0.8 W cm^2^).

In the treatment of gastric cancer, Li et al. (2016) used aloe emodin to fabricate aloe emodin nanoliposomes for transfection of the r-caspase-3 gene and PDT. Moreover, hypericin is an excellent photosensitizer in hypericin–low-density lipoprotein–dextran complexes for glioma treatment. In these complexes, dextran is modified on the surface of low-density lipoprotein particles, which reduces the interaction of low-density lipoprotein with other serum constituents to prevent hypericin redistribution to other free lipoproteins (Huntosova et al., 2012).

Also, single-walled carbon nanohorn-hypericin has been shown to improve the water solubility, photostability, and therapeutic effects of hypericin and to protect it from light degradation. Single-walled carbon nanohorn-hypericin can simultaneously generate sufficient ROS and hyperthermia upon light irradiation at 590 and 808 nm (Gao et al., 2019a).

### Thermal-Responsive NFs for Delivery of Active Compounds From TCM

In thermal-responsive chitosan-g-poly(*N*-isopropylacrylamide) co-polymeric nanoparticles, the polymer–polymer interaction of poly(*N*-isopropylacrylamide) is greater than the polymer-curcumin interaction, whereas the hydrogen bond is weakened at a lower critical solution temperature, so curcumin molecules can escape from the entrapped polymer matrices (Rejinold et al., 2011). Honokiol hydrogel (Fang et al., 2009) loads honokiol as a free-flowing sol at room temperature or below the critical gelation temperature. Honokiol can become a gel at body temperature and remain *in situ* for a long time to reduce the burst release of honokiol.

## Multiple-Responsive NFs for Delivery of Active Compounds From TCM

Multiple-responsive NFs usually have two or more different responsive functions and modes. The combination of multiple responsive modes can overcome the shortcomings of a single responsive mode to achieve targeted delivery of drugs. The main multiple-responsive NFs are shown in Table 4.

**Table 4:** Multistimulus-responsive nanoformulations in active compounds from TCM.

**Response method**	**Active compounds**	**Response agent**	**Application**	**Cell line/animal**	**Reference**
pH and redox	Berberine	pH: vitamin B_6_ redox: dithiodipropionic acid	Anticancer	HepG2 HepG2 tumor-bearing nude mice	Fang et al., 2019a
	Berberine and curcumin	pH: borate ester bond redox: dithiodipropionic acid	Anticancer	PANC-1 PANC-1 tumor-bearing nude mice	Fang et al., 2019b
	Celastrol	pH: tertiary amines Redox: disulfide bond	Desmoplastic melanoma (DM)	BPD6 NIH 3T3 DM tumor-bearing mice	Liu et al., 2018
	Cinnamaldehyde	Acetal and quinone methide	Colon cancer Breast cancer	CT26 4T1 CT26 tumor-bearing mice	Ma et al., 2020
		Redox: boric acid pH: acetal linkage	Prostate cancer Colon cancer	DU145 SW620 NIH3T3 BALB/c nude mice	Noh et al., 2015
	Curcumin	Redox: dithiodipropionic acid pH: hydrazone	Multidrug-resistant	MCF-7/ADR	Wang Y. et al., 2018
		pH: imine bond Redox: disulfide bond	Anticancer	Hep3B HA22T/VGH	Massaro et al., 2016
		pH: calcium phosphate and ester bond Redox: disulfide bond	Anticancer	MCF-7 A549 MDA-MB-231 BALB/c mice	Chen et al., 2017
	Podophyllotoxin	Redox: disulfide bond pH: disulfide bond and b-thiopropionate	Breast cancer	MCF-7 MCF-7/ADR A549 A549/PTX MCF-7/ADR tumor-bearing nude mice	Li et al., 2019b
Magnetic and pH	Artemisinin	Magnetic: Fe_3_O_4_@C pH: MIL-100(Fe)	Anticancer	HeLa A549 BALB/c nude mice	Li et al., 2019a
	Berberine	Magnetic: Fe_3_O_4_ head pH: carboxylate functional group modified on the surface and pore of silica rod	Hepatocellular carcinoma	HepG2 HL-7702	Wang M. et al., 2017
	Berberine and doxorubicin	Magnetic: Janus M-MSNs pH: Janus M-MSNs	Hepatocellular carcinoma	H22 HepG2 NIH-3T3 HL-7702 H22 tumor-bearing ICR mice	Zhang et al., 2019
	Quercetin	Magnetic: Fe_3_O_4_@SiO_2_ (FITC) pH: imine and acetal	Multidrug-resistant	A549 A549/DOX	Daglioglu, 2017
Magnetic and thermal	Stevioside	Fe_3_O_4_	Glioma	C6	Gupta and Sharma, 2019
Thermal and pH	Cinnamaldehyde	Thermal: glycine and Pluronicpolymer pH: Fe_3_O_4_ nanoparticles	Breast cancer	MCF7 MDAMB231	Wani et al., 2014
	Berberine and evodiamine	Thermo: *N*-isopropylacrylamide pH: methacrylic acid	Anticancer	HepG-2 HCT-8 HeLa HUVEC EMT-6 tumor-bearing nude mice	Feng et al., 2019
Light and redox	Curcumin	Zinc phthalocyanine and curcumin	Melanoma tumor	B16F10 mouse	Zhang et al., 2020b
Magnetic and light and thermal and pH	Artemisinin	Magnetic and photothermal: PB MOF Magnetic and PH: MIL-100(Fe) MOF	Anticancer	HeLa BALB/c nude mice	Li et al., 2019a
Magnetic and light and thermal	Gallic acid	Magnetic and thermal: SPIONs Light: ICG	Anticancer	MCF7 HT-29 4T1 tumor-bearing mice	Jin et al., 2017
Light and thermal and pH	Quercetin	pH: poly(ethylene glycol)_5k_-poly(β-aminoesters)_10k_ Light and thermal: Ag_2_S	Anticancer	HepG-2 HL-7702 HepG-2 tumor-bearing nude mice	Zhong et al., 2020

### Dual Internal–Responsive NFs

For targeted delivery of cinnamaldehyde, quinone methide-cinnamaldehyde (Noh et al., 2015) was prepared as a dual-responsive NF combining a redox response and pH response. The H_2_O_2_-sensitive boronate linkage and acid-sensitive acetal linkage were oxidized rapidly to release quinone methide to abrogate GSH. Also, the acetal linkage degraded in acidic tumor environments to release ROS-generating cinnamaldehyde, which killed DU145 and SW620 cells efficiently *in vitro* and *in vivo*. The same type of response mode was used in tumor-specific enhanced oxidative stress polymer conjugate (Ma et al., 2020) delivery of cinnamaldehyde on CT26 and 4T1 cells. The acidic condition accelerated hydrolysis of acetal linkages in phenylboronic acid containing cinnamaldehyde derivatives, and H_2_O_2_ facilitated detachment of boric acid groups and intramolecular rearrangement to produce quinone methide and further accelerated the hydrolysis of acetal linkages and cinnamaldehyde release.

Celastrol was delivered to desmoplastic melanoma cells by aminoethylanisamide–polymer–disulfide bond nanoparticles (Liu et al., 2018). In an acidic tumor microenvironment, tertiary amines of mitoxantrone and celastrol copolymer were protonated and reversed the zeta potential from a negative charge to a positive charge, thereby attracting negatively charged cell membranes. Also, the disulfide bond was broken in the presence of GSH, resulting in an increased particle size.

Disulfide bonds and β-thiopropionate linkages endowed a redox response and pH response of T7-peptide-S-S-nanoparticles (Li et al., 2019b) for POD delivery. Disulfide bonds were disassociated by a high concentration of GSH, and the β-thiopropionate linkage was broken by an acidic environment, and then POD was released rapidly. Massaro et al. replaced the keto group of curcumin with a pH-responsive imine group and used a carbon chain with a disulfide bond to connect the silicon on halloysite nanotubes to prepare a pH/redox responsive curcumin prodrug. In the different pH and 10 mM GSH environment, it showed a good release behavior of curcumin (Massaro et al., 2016).

### External/Internal–Responsive NFs

Multiple-responsive NFs with both external and internal responses are conducive to targeted release of TCM agents at lesion sites. As stated above, often magnetically responsive delivery is combined with other responsive modes for higher efficiency of drug delivery.

A multiple-responsive NF called Prussian blue-iron carboxylate dual-metal organic frameworks [PB@MIL-100(Fe)dual-MOFs] can deliver artemisinin to HeLa cells through integrated magnetic, light and pH responses (Wang et al., 2016b). The inner Prussian blue MOFs and outer MIL-100(Fe) MOFs serve as MRI contrast agents. Under the illumination of an 808-nm near-infrared laser, the inner Prussian blue MOF induces hyperthermia by converting light energy to heat. The MIL-100(Fe) MOF can collapse in acidic environments to release artemisinin.

Dihydroartemisinin was delivered using a strategy combining a magnetic response and pH response. Fe_3_O_4_@C@MIL-100(Fe) nanoparticles were designed by Wang and colleagues (Wang et al., 2016a). Fe_3_O_4_@C nanoparticles were magnetic targeting, and carbon dots were two-photon fluorescence imaging agents. MIL-100(Fe) MOFs degraded and released dihydroartemisinin and Fe (III) ions in acidic conditions. When the pH within an endosome decreased, Fe^3+^ reduced to Fe^2+^ by ferric reductase and other reductive molecules within cells.

Magnetic field–induced endocytosis and pH-responsive drug release of berberine-loaded Fe_3_O_4_-mSiO_2_ nanoparticles were exhibited in hepatocellular-carcinoma treatment because the pH-responsive carboxylate functional group was modified on the surface and pores of silica rods and the magnetic-responsive Fe_3_O_4_ head (Wang Z. et al., 2017). In stevioside (STE)-MNP, STE reduced the size of Fe_3_O_4_ nanoparticles to control magnetic properties and aligned rapidly with the external magnetic field (Gupta and Sharma, 2019). The carbohydrate nature of STE enhanced the interaction between STE-MNP and C6 cells. STE-MNP aggregated as cluster-like formations in the glioma site to enhance hyperthermia.

The magnetic-responsive agents, superparamagnetic iron oxide nanoparticles (SPIONs), and a light-responsive agent, indocyanine green (ICG), were combined in SPIONs-GA-ICG (Ghorbani et al., 2018). The SPIONs produced hyperthermia; gallic acid was oxidized and bonded to ICG by free-radical production, and gallic acid also acted as a biological coating for ICG-loaded SPIONs.

A thermal- and pH-responsive NF was used as a cinnamaldehyde-delivery platform with glycine and a pluronic polymer. Cinnamaldehyde tagged Fe_3_O_4_ nanoparticles capped with glycine and pluronic polymer nanoparticles increased the temperature to 41.6°C within 1 min after radiofrequency pulses of 20 MHz (Wani et al., 2014). Also, the ionic interaction between the free aldehyde group of cinnamaldehyde and the liberated protons under acidic conditions delivered cinnamaldehyde rapidly from the nanoparticles.

## Summary

Most of the active compounds extracted from TCM are small molecules. They have unfavorable pharmacokinetics and suboptimal biodistribution (e.g., prominent accumulation in multiple healthy organs) (Golombek et al., 2018). Through nanotechnology modification, drug molecules have a better EPR effect to increase the accumulation of drugs at lesion sites (Maeda, 2017). Thus, several types of nanodelivery system have been employed to improve the clinical outcome of the active compounds in TCM formulations: nanoparticles, liposomes, micelles, nanocapsules, and nanoemulsions (Ma et al., 2019).

Stimuli-responsive NFs can release drugs under internal and external stimuli. We reviewed the applications of stimuli-responsive NFs used commonly in the delivery of active compounds from TCM.

Four types of pH-responsive agents can be classified. The first type is inorganic material. Iron-containing nanoparticles play a key part in the pH-responsive delivery of artemisinin. The endoperoxide linkages (R-O-O-R′) in artemisinin can be cleaved with intracellular Fe^2+^ to generate toxic radicals to kill cancer cells and treat malaria (Asano and Iwahashi, 2017; Ding et al., 2018). Fe_3_O_4_ dissociates rapidly at low pH in lysosomes and transforms to Fe^2+^ (Bhaw-Luximon and Jhurry, 2017). Simultaneously, Fe_3_O_4_ is also a magnetic agent that can induce a magnetic response. In Fe_3_O_4_@MnSiO_3_-FA nanospheres, Mn^2+^ has the same function as Fe^2+^ (Chen et al., 2015). ZIF-8 is a well-known pH-sensitive imidazole derivative (Chen et al., 2018; Gao et al., 2019b) composed of Zn^2+^ coordinated by four imidazolate rings (Mateo et al., 2019). ZnO nanoparticles are also degraded readily to Zn^2+^ (Hahn and Ahmad, 2012). ZnO is stable at pH ~7, but dissolves rapidly at pH < 6 (Dhivya et al., 2018). The second type of pH-responsive material is a natural product with pH instability. Chitosan is a commonly used drug-delivery agent with special amphiphilic characteristics that enables “packaging” of a poorly soluble drug in water phases (Younes, 2015). Its pH-responsive function is attributed mainly to protonation of amino groups in acidic conditions, which make chitosan hydrogels swell rapidly and release the loaded drug. The third type of pH-responsive materials is artificial polymers such as PAE, PAGA, and PLGA. The fourth (and most common) types of pH-responsive material used in the delivery of active compounds from TCM are amine bonds and hydrazone bonds. They are linked ingeniously with the active agent from the TCM and the matrix of NFs until they encounter an acidic microenvironment. This strategy protects the drug from leaking prematurely in the circulation and promotes the targeted release of the drug at the lesion site.

Redox-responsive NFs of active compounds from TCM are dependent mainly on modification of disulfide bonds. Metabolic imbalances in tumor cells produce high levels of GSH, which can cleave disulfide bonds to release the loaded agent in target cells. In addition, pathogen infection induces an inflammatory reaction to increase H_2_O_2_ levels in infected tissues, which are prone to redox reactions.

In light-responsive NFs, besides the many chemical photosensitizers, several naturally active ingredients of TCM are used as photosensitizers in PDT, including curcumin (Tao et al., 2019b), hypericin (Lin et al., 2016; Li et al., 2018b; Zhang et al., 2018a), and aloe emodin. These active compounds from TCM have been shown to be useful in PDT or PTT in multiple studies.

Some active substances from TCM have been used for nanotargeted delivery. In doxorubicin-loaded CTS/PEG-GA nanoparticles (Tian et al., 2010), GA can target GA receptors on liver cancer cells. Also, GA receptors have been shown to be useful as liver-cancer targets in GA NFs (Sun et al., 2017). Similarly, in quercetin/Fe (III) (Bertleff-Zieschang et al., 2017) delivery, quercetin (as a phenolic compound) can form a complex organic–inorganic network with iron and gallic acid, and STE contributes to regulation of iron ions to form an “ideal” size of magnetic nanoparticles. Some active compounds from TCM contain both water-soluble sugar groups and water-insoluble aglycones, which form biocompatible NFs readily by self-assembly. Also, most active compounds from TCM have multiple active binding sites in biological tissues.

In stimuli-responsive NFs, internal stimulus–responsive NFs have great advantages in specifically releasing active compounds from TCM to the target site, subsequently increasing their therapeutic efficacy and decreasing the side effects. However, it is very difficult to artificially control the amounts and time of drug release from internal stimulus–responsive NFs. External stimulus–responsive NFs have advantageous properties in artificial control of drug release because they could respond to external light and magnetic energy. Therefore, we could regulate light and magnetic energy to control the release amounts and time of drugs from these NFs.

The information provided above indicates that smart responsive NFs hold great promise in targeted delivery of active compounds from TCM. However, the confirmed evidences are mainly from *in vitro* and *in vivo* experimental studies. Few clinical trials are investigated on smart responsive NFs as the targeted carriers for delivering active compounds from TCM. The main challenges are the complexity of smart responsive NFs such as tedious preparation, complicated characterization, biosafety of nanoparticle materials, and uncertainty of the *in vivo* fate of NFs. Thus, further exploring and addressing the above problem should be urgent tasks for translating smart responsive NFs as targeted delivery carriers of active compounds from TCM to clinical application.

## Author Contributions

All authors listed have made a substantial, direct and intellectual contribution to the work, and approved it for publication.

## Conflict of Interest

The authors declare that the research was conducted in the absence of any commercial or financial relationships that could be construed as a potential conflict of interest.
